# Rolling Circle Amplification as a Molecular Tool for Spatially Resolved Signal Amplification in Single Molecule Counting Assay

**DOI:** 10.3390/bios15090628

**Published:** 2025-09-21

**Authors:** Juhwan Park

**Affiliations:** Department of Bio and Fermentation Convergence Technology, Kookmin University, Seoul 02707, Republic of Korea; juhwan@kookmin.ac.kr

**Keywords:** rolling circle amplification, signal amplification, single cell, single extracellular vesicle, single molecule counting assay

## Abstract

There have been rising interests in ultra-sensitive biosensing technologies for early diagnosis and prognosis monitoring of infectious diseases, cancers, and neurodegenerative diseases. Digital signal readout strategy represented by digital ELISA or digital PCR, advanced biosensing field enormously, which enables detection of biomolecules under the detection limit of conventional biosensing methods. However, due to the need for compartmentalization and limited multiplex capability, it has been hurdled for utilization in applications requiring hierarchical resolution analysis such as sub-cellular molecules or molecular cargo of single cells or single extracellular vesicles (EVs). Rolling circle amplification (RCA), an isothermal DNA amplification method enabling localization of an amplified signal, can eliminate the need for compartmentalization and increase multiplex capability. It also has potential to expand applications of single molecule counting assay for understanding hierarchy of biological systems. In this review, recent advances in RCA-based single molecule counting assay are overviewed and their applications in single cells and single EVs quantitative analysis are discussed. Furthermore, the limitations and outlook of RCA-based single molecule counting assay are highlighted.

## 1. Introduction

Due to the increasing importance of ultra-sensitive biosensing technologies for diseases prognosis and diagnosis in infectious diseases, cancers, and neurodegenerative diseases, numerous ultra-sensitive biosensing tools have been developed, including fluorescence, nanoplasmonic, and electrochemical sensing [1,2,3,4]. Single molecule counting assay based on digital signal readout strategy has been emerged for ultra-sensitive detection of various biomarkers [5,6]. Notably, digital ELISA represented by single molecule array technology (SiMOA) technology commercialized by Quanterix has been widely adopted due to its automated system and robustness [7,8]. SiMOA technology has been applied to detect clinically relevant biomarkers including phosphorylated tau 217 (p-Tau 217) [9], inflammatory markers [10], and extracellular vesicles’ surface protein [11]. Digital ELISA utilizes enzyme-based signal amplification, which requires compartmentalization of immunocomplexes by water–oil interfaces to localize amplified fluorescence signals [12,13].

On the other hand, digital signal readout strategy for nucleic acid detection, first introduced by droplet digital polymerase chain reaction (PCR), has also gained significant attention. Encapsulation of DNA/RNA with amplifying reagent within water-in-oil droplet followed by thermal cycling generates fluorescence signal in droplets having target nucleic acids [14]. Isothermal amplification strategies such as loop-mediated isothermal amplification (LAMP), nucleic acid sequence-based amplification (NASBA), and recombinase polymerase amplification (RPA) have been developed as digital assay platforms using microwells or droplet microfluidics [15,16,17,18]. Recently, the CRISPR/Cas system has also been employed for rapid detection of nucleic acid at the single molecule level by using compartmentalization strategy, where the fluorescence signal is amplified by cleaving DNA probe that bridge a quencher and fluorophore in the presence of target sequence [19,20].

**Figure 1:**
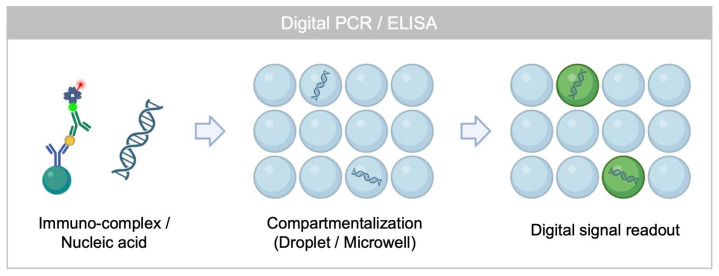
Working principle of conventional single molecule counting assay based on digital signal readout strategy.

Although numerous digital assays have enabled ultra-sensitive biosensing, their reliance on compartmentalization make assay procedures complicated (Figure 1) [21]. Additionally, enzyme-based signal amplification limits the multiplexed analysis, as there are few stochiometric combinations of enzymes and substrates to amplify fluorescence signal in differentiated excitation and emission spectrum [22], although PCR or CRISPR/Cas system have overcame the limitation in multiplexed capacity by employing detection probes conjugated with different fluorescent dyes [23,24]. Moreover, conventional single molecule counting assay based on compartmentalization limit hierarchical analysis of biomolecules (e.g., single molecule level profiling of single cell or EV) because amplified signal is not spatially localized.

Rolling circle amplification (RCA), an isothermal DNA amplification method inspired by the bacterial plasmid replication mechanism, has been fascinating for a few decades in the biosensing area [25]. With DNA primer, circle DNA, dNTPs, and enzyme, DNA primer is elongated along circle DNA (5′ to 3′) forming aggregated DNA nanoparticles [26,27]. To form a circle DNA hybridized with primer, padlock probes can be hybridized and ligased [28], or pre-circularized DNA by Circligase can be hybridized to a primer [29]. For RCA, phi29 DNA polymerase, showing optimal activity near 30 °C, is the most widely used enzyme, as it is capable of displacement synthesis with 3′ to 5′ exonuclease activity and high fidelity [30].

**Figure 2:**
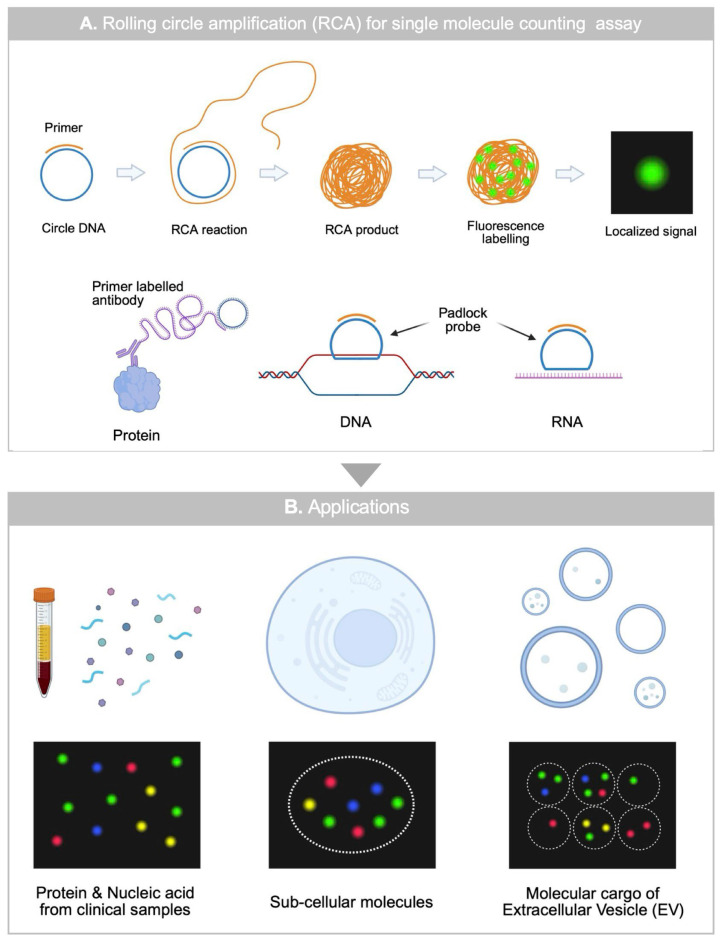
An overview of RCA-based single molecule counting assay (**A**) Working principle of RCA-based single-molecule counting assay for protein, DNA, RNA. (**B**) Applications of RCA-based single molecule counting assay for analyzing clinical samples, single cells, and single EVs.

RCA can generate repeated DNA sequences, making it an attractive method to amplify the signal of target biomolecules, which is applicable for both nucleic acids and protein detection (Figure 2A) [31,32,33]. Unlike enzyme-substrate reaction-based signal amplification producing freely diffusing fluorescence molecule, RCA generates aggregates of repeated DNA sequence tethered to the site of the circle DNA. These products can be labeled with complementary fluorescent probes, enabling localized signal amplification and preservation of spatial information.

RCA can further improve the performance of the bioassay by synergetic with single molecule counting assay based on digital signal readout by excluding the needs for compartmentalization. Furthermore, RCA can be applicable for quantitative analysis of cells or EVs, as it can spatially resolve biomarker expression under sub-cellular level (Figure 2B). Here, we report a recent advances in RCA-based single molecule assays and its applications for single-cell and single-EV quantitative analysis. Moreover, we discuss outlook of RCA based single molecule assay towards ultra-sensitive and multiplexed single-molecule level biosensing applications.

## 2. RCA-Based Single Molecule Assay for Protein and Nucleic Acid Detection from Clinical Specimen

As RCA can localize the amplified signal from a target protein, RNA or DNA, it has been advanced to a digital assay platform enabling counting of molecules. To apply RCA on protein detection via immunoassay, DNA primer is conjugated to the antibody using a click chemistry such as DBCO-Azide or TCO-Tetrazine [34]. Like ELISA, primer conjugated detection antibody labels captured antigens on a substrate or microbead, and then conjugation of circle DNA and addition of RCA mixture initiate elongation of ssDNA. Circle DNA can be pre-formed from ssDNA by Circligase or it can be formed after the hybridization and ligation of padlock probe to the primer. Although Circligase can simplify the assay workflow, its high cost and relatively lower efficiency compared to conventional ligases remain a limitation. After the RCA reaction under DNA polymerase such as phi29 at the reaction condition, labeling of elongated ssDNA with fluorescent detection probe allows visualization of RCA products that indicate the location of captured antigen. RCA is also applicable for the detection of DNA and RNA, which are captured on a substrate or microbead by using a complimentary DNA probe for further hybridization with circle DNA for RCA reaction. After the hybridization of target DNA or RNA, conjugation of circle DNA can initiate RCA reaction enabling localization of amplified signal after the fluorescence detection probe labeling.

Compared with conventional PCR, RCA amplifies the initial DNA target in a linear rather than exponential manner, leading to slower amplification. Nevertheless, the localized nature of RCA amplicons provides significant advantages for single-molecule counting assays. RCA efficiency depending on various factors has also been assessed, including the length of circle DNA, PEG concentration in RCA mixture, and CA contents in circle DNA [35,36]. Due to the steric hindrance of circle DNA, it shows cyclic characteristics on RCA efficiency on every 10.2 nucleotide length [36]. Increased PEG concentration provided a molecular crowding environment enabling highly efficient complex formation between enzyme and RCA templates, resulting in increased fluorescence intensity of RCA products [35].

### 2.1. Protein Detection

**Figure 3:**
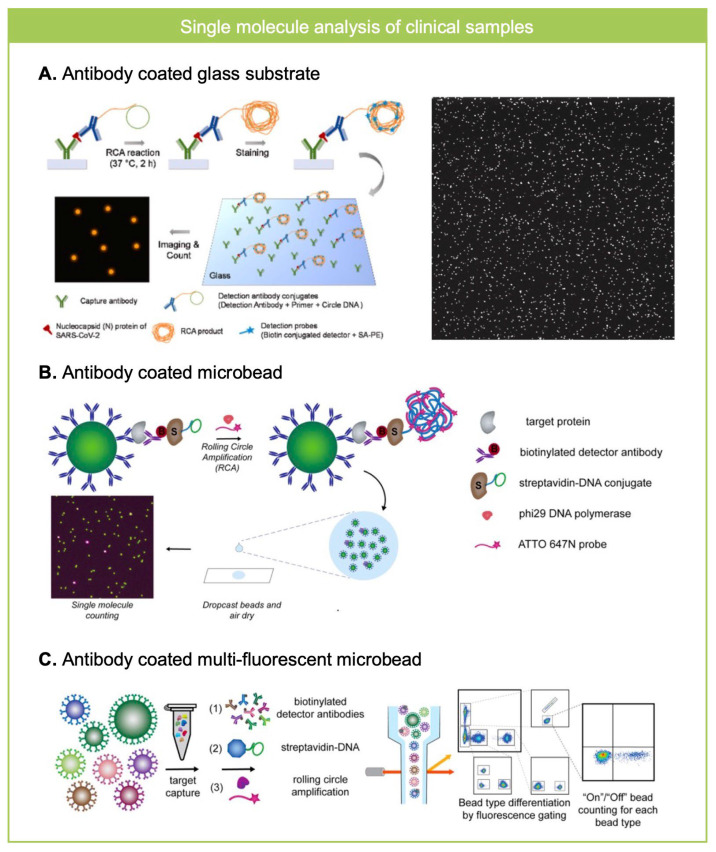
RCA for single molecule counting assay in clinical samples. (**A**) Nucleocapsid (N) protein of SARS-CoV-2 from saliva sample was analyzed where N protein was captured on antibody coated glass substrate and was labeled with RCA template and imaged after the RCA reaction and fluorescence labeling. Reprinted/adapted with permission from Ref. [37]. Copyright 2023, Elsvier. (**B**) Antibody-coated microbeads were used to analyze Brachyury, a T-box transcription factor from plasma sample by using RCA based single molecule counting assay. The ratio of microbeads co-localized with RCA product was measured, which shows higher analytical performance than SiMOA. Reprinted/adapted with permission from Ref. [41]. Copyright 2020, American Chemical Society. (**C**) Multi-fluorescent antibody coated microbeads were used to analyze eight different cytokines from plasma or saliva samples using RCA-based single molecule assay. Flow cytometry based high-throughput readout enables decrease in background noise so the revealed ultra-sensitive and multiplexed measurements of biomarkers from clinical specimens. Reprinted/adapted with permission from Ref. [40]. Copyright 2022, American Chemical Society.

Since RCA can localize the amplified signal with high SNR, it has been implemented for ultra-sensitive detection of biomolecules from clinical specimens. Park et al. detected nucleocapsid (N) protein of SARS-CoV-2 from human saliva sample with >100 times improved sensitivity compared to conventional ELISA (Figure 3A) [37]. They utilized glass substrate to immobilize capture antibody and immune-RCA assay for the localization of an amplified signal from a captured antigen. PDMS microwell was sealed on a glass slide to form assay spots and spots were modified with capture antibody by (3-Aminopropyl)triethoxysilane (APTES)-glutaraldehyde reaction. By counting the absolute number of fluorescent punctate on a glass substrate, as low as <1 pg/mL of SARS-CoV-2 N protein was detected distinguishing COVID-19 patients with 99.5% specificity and 90.9% sensitivity. By counting the number of RCA products in the analysis region, the amount of antigen can be analyzed at the single molecule level that visualizes the location of the target analyte [38]. Based on a theoretical study of antigen–antibody binding kinetics, a single molecule counting assay can ideally quantify antigens down to the attomolar level by assuming there is no non-specific binding and diffusion limit of biomolecules [37,39]. Nevertheless, due to the non-specific binding and diffusion limit of biomolecules, single molecule counting assay on a glass substrate revealed that antigens have been detected as much as the femtomolar level [37].

Diffusion limit of biomolecules has been addressed by using capture antibody conjugated magnetic microbead instead of using capture antibody coated substrate (Figure 3B,C) [40,41]. By counting the ratio of microbead co-localized with RCA products, antigen was detectable at a sub-femtomolar concentration, achieving 25-fold improved sensitivity compared to SiMOA technology. By loading the dropcast of beads on a glass substrate after the RCA reaction and fluorescence labeling, it was imaged to count the positive ratio of beads co-localized with fluorescently labelled RCA products. They demonstrated that an increase in the number of beads analyzed can improve the limit of detection by decreasing the Poisson noise. By using their RCA-based single molecule assay platform, Brachyury, a T-box transcription factor from a plasma sample was successfully detected over the noise floor of the assay where SiMOA was not able to detect. In addition to overcoming a diffusion limit, improved LOD with a decrease in background noise was achieved by analyzing microbeads >10,000 to decrease Poisson noise (1/sqrt (N)) < 1% via flow cytometry [40]. Flow cytometry enabled high-throughput analysis of microbeads and RCA products co-localized on them. By using fluorescent encoded beads, multiplexed RCA-based single molecule counting assay was utilized to detect eight different cytokines from plasma or saliva samples. Multiplex assay has also been achieved by using different sequence of circle DNA in substrate based RCA assay [35]. By using multiple capture antibodies, detection antibodies with different primers, circle DNA and fluorescent detection probes, multiple antigens, or nucleic acid can be detected at the same time.

### 2.2. Nucleic Acid Detection

Without substrate or microbead, RCA can be performed in solution where RCA products are labeled with detection probe directly added in solution. Fluorescently labeled RCA products are concentrated on filter-incorporated microfluidic channel or nitrocellulose membrane while unbound detection probes are washed out [42,43]. Using this method, multiplexed detection of Ebola, Dengue, and the Zika virus were demonstrated by using different padlock probes and fluorescent detection probe in solution phase [43]. By sequentially adding the reagents for ligation, RCA, followed by their stepwise inactivation, successful detection of viral genomic material was achieved.

## 3. RCA for Single Molecule Analysis of Single Cells

Single cell analysis has emerged with advances in micro/nano technologies, such as droplet microfluidics and sequencing technologies [44,45]. Due to the heterogeneity of single cells, it has been necessary to identify different subpopulation of cells from tissue sample (e.g., biopsy tissue sample) or body fluid (e.g., circulating tumor cells). Even though sequencing technologies combined with droplet microfluidics like Drop-seq [46], Indrops [47], or cellular indexing of transcriptomes and epitopes by sequencing (CITE-seq) [48] have provided detailed information regarding mRNA and surface protein expression in single cell level with high-throughput, it has been difficult to utilize them as a translational research tool due to the need for complicated downstream analysis procedures for sequencing and high cost. Therefore, there has been an increasing need for platform technologies to quantitatively profile interesting molecules within single cells. Additionally, there is a great interest in spatial information of transcriptomes within individual cells to infer high-dimensional biological evidence [49,50]. RCA offers an appropriate solution for these needs that can amplify the signal from target molecule and localize amplified signals at the position of the target molecules [51]. Moreover, it can encode distinct fluorescence signals for multiple targets and is applicable to both proteins and nucleic acids, offering significant advantages for multiplexed and multi-modal assays.

### 3.1. Protein and RNA

**Figure 4:**
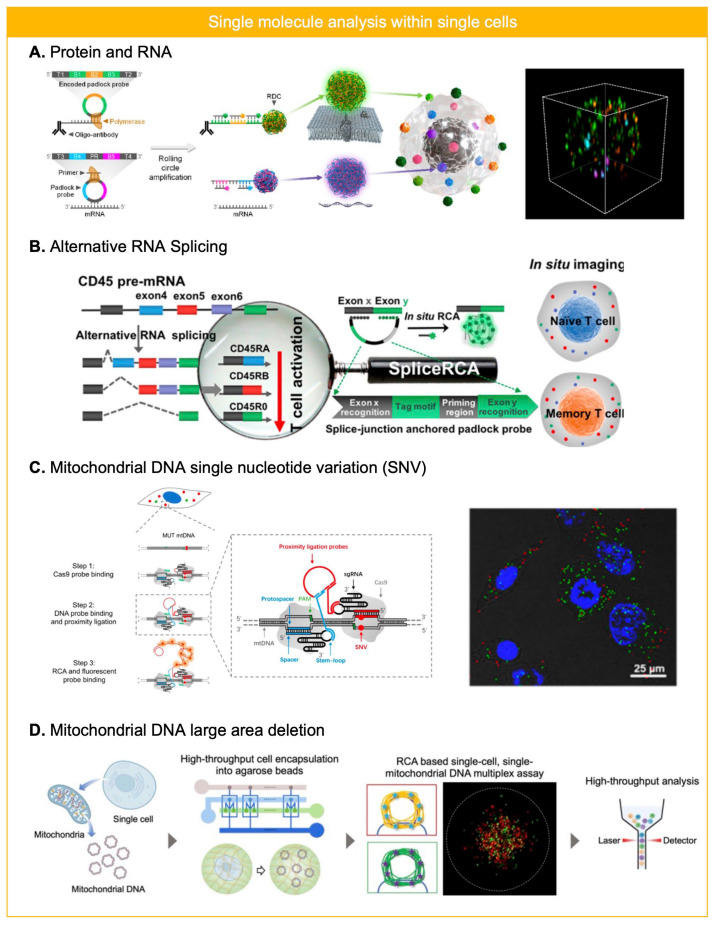
RCA for single molecule analysis in single cells. (**A**) Protein and RNA were analyzed simultaneously within fixed and permeabilized cells on a coverslip by employing primer conjugated antibody and padlock probe. Reprinted/adapted with permission from Ref. [53]. Copyright 2024, American Chemical Society. (**B**) Alternative mRNA splicing was analyzed for naïve or memory T cell by using padlock probe based in situ RCA. Reprinted/adapted with permission from Ref. [54]. Copyright 2018, Americal Chemical Society. (**C**) Single nucleotide variation (SNV) of mitochondrial DNA (mtDNA) was profiled in single cell level by utilizing CRISPR/Cas based recognition and RCA reaction after the proximity ligation. Reprinted/adapted with permission from Ref. [65]. Copyright 2018, American Chemical Society. (**D**) mtDNA large area deletion was profiled by padlock probe-based RCA assay in single cell level. By trapping mtDNA from single cells within agarose microbeads, two different regions of mtDNA were targeted using different padlock probes to assess heteroplasmy of mtDNA large area deletion. RCA products within agarose microbeads enabled flow cytometry based high-throughput readout. Reprinted/adapted with permission from Ref. [56]. Copyright 2024, John and Wiley Sons.

Deng et al. reported DNA-sequence encoded barcoding based RCA for single cell RNA analysis, which can provide spatial information of RNA in single cells [52]. After the fixation and permeabilization of cells on a coverslip, mRNA within cell is targeted by padlock probe, and additional primer was hybridized to the ligated padlock probe for RCA reaction. RCA reaction elongates complementary sequence of ligated padlock probes, which can be labeled with fluorescent detection probes. By designing a padlock probe with a different ratio of sequences for fluorescent probe hybridization, multiplex capacity of single cell RNA profiling increased up to nine for single cell RNA profiling. Furthermore, for simultaneous profiling of both protein and transcriptomes within individual cells, Shin et al. developed RCA based multi-modal single cell analysis platform (Figure 4A) [53]. On permeabilized cells fixed on a glass slide, simultaneous labeling of protein and transcriptome enabled identification of oncogenic transcript subtypes in B-cell acute lymphoblastic leukemia. Primer conjugated detection antibody was used for protein labeling and padlock probe and primer was used to formulate RCA product from mRNA. Three-dimensional imaging of RCA products within single cells revealed 5-plex analysis of RNA (2-plex) and protein (3-plex).

mRNA splicing variants of immune cells were profiled in single cell level using RCA-based single molecule assay, which they call splice-junction anchored padlock-probe-mediated RCA assay (Figure 4B) [54]. By targeting two exon regions of CD45 mRNA using padlock probes, three different isoforms of CD45 mRNA were analyzed at a single cell level where differently encoded RCA products were generated depending on how RNA splicing was happened to form CD45 mRNA that can be used to understand T cell activation.

RCA-based single cell analysis has been further progressed coupled with droplet microfluidic technologies. Although substrate-based imaging methods can analyze fixed and permeabilized cells, droplet microfluidics can improve the throughput critical to identify rare population of cells. Water-in-oil droplets have advanced the single cell analysis technologies and the user-interface has been further improved by water-in-oil-in-water (W/O/W) double emulsion droplets, which is compatible with flow cytometry or FACS [55]. Recently, hydrogel droplet microfluidic technologies have provided advantages for single cell analysis over W/O or W/O/W droplets [56,57]. Hydrogel droplets are also compatible with a conventional high-throughput analysis tools (Flow Cytometry or FACS) and they can be processed in bulk without manipulation in a single droplet level [58], as it can retain or conjugate biomolecules to their porous networks. In this manner, RCA-based single molecule analysis has been coupled with hydrogel droplet microfluidics for single cell analysis, where RCA has been compatible with hydrogel particles [59,60]. Rakszewska et al. encapsulated single cells within hyaluronic acid (HA) droplets modified with capture probe, and transcripts from single cells are captured to porous structure of HA droplets [61]. HA has been used as a base material due to its chemical versatility and anti-fouling property to increase signal-to-noise ratio [62]. After the gelation of HA hydrogel, reverse transcription of transcripts, hybridization and ligation of padlock probe, and RCA reaction visualized signal from the captured transcripts for single cell level quantification.

### 3.2. Genomic Material of Sub-Cellular Organelle

Besides transcriptome and protein, genomic material of sub-cellular organelle such as mitochondria can be investigated by employing RCA-based single molecule assay. The mutation in mitochondrial DNA (mtDNA), including large area deletion and single nucleotide variation (SNV), has been known to be correlated with metabolism-related diseases such as diabetes, sarcopenia, and cancers [63,64]. In order to screen SNV of mtDNA, CRISPR/Cas 9 recognition-initiated RCA-based assay has been developed by utilizing fixed cells on a glass substrate. As SNV shows high inter- and intra-cellular heterogeneity, accurate recognition characteristics of CRISPR/Cas systems were employed, and then proximity assay-based RCA reaction was performed to assess the presence of SNV within cells (Figure 4C) [65]. By comparing the ratio of differently encoded RCA products within cells, intra cellular heterogeneity of single cells can be validated, while inter-cellular heterogeneity can be validated by comparing the ratio of RCA products between different cells.

An agarose droplet-based single-cell, single-mtDNA large area deletion screening platform has been developed by utilizing a padlock probe-based RCA assay (Figure 4D) [56]. By trapping the large sized (16 kb) mtDNA within the porous structure of agarose, single-cell mtDNA large area deletion was analyzed without cross-contamination between agarose beads. Agarose has been used because it possesses a permeable pore size on the order of hundreds of nanometers, readily gels below its gelling temperature (~20 °C), and exhibits no cytotoxicity. By targeting the deletion region with the padlock probe after the denaturation of mtDNA using DMSO, ligation of padlock probes and annealing of primer initiated RCA reaction to formulate large DNA particle within agarose beads. By targeting two different regions, one for the conserved and the other for the detectable region, the amount of mtDNA large area deletion was analyzable at the single cell level.

### 3.3. Secreted Molecules

In addition to molecules within cells, secreted molecules from cells such as immunoglobulin (Ig) can be analyzed by using RCA [66]. B cells are compartmentalized into microwells and they were covered by an antigen-coated coverslip that can capture the secreted Ig from an activated B cell within microwell. Secreted Ig captured on the coverslip were labeled with primer-conjugated detection antibodies targeting three different antigen-specific antibody isotypes (IgA, IgG, and IgM). After the conjugation of padlock probe, ligation, and RCA reaction, isotypes of antibodies secreted from single cells were quantified by analyzing the number of different fluorescence signals.

## 4. RCA for Single Molecule Analysis of Single EVs

Recently, the importance of single EV profiling has been emerged due to their high heterogeneity in clinical samples to understand EV’s origin and roles [67,68]. As single EV has >100× smaller size than single cell, corresponding to 10,000× smaller surface area and 1,000,000× smaller volume than cell, supposing much less protein and RNA expression level. Therefore, there is much higher need for ultra-sensitive molecular profiling of single EVs for both surface protein and internal RNA. Although there have been notable advances in fluorescence-based methods for EV profiling including ultra-sensitive flow cytometry [69,70] and ultra-sensitive imaging tool like total infrared fluorescence microscope (TIRF) [71], it still requires higher sensitivity to profile single-molecule level cargo of single EVs.

### 4.1. Surface Protein

**Figure 5:**
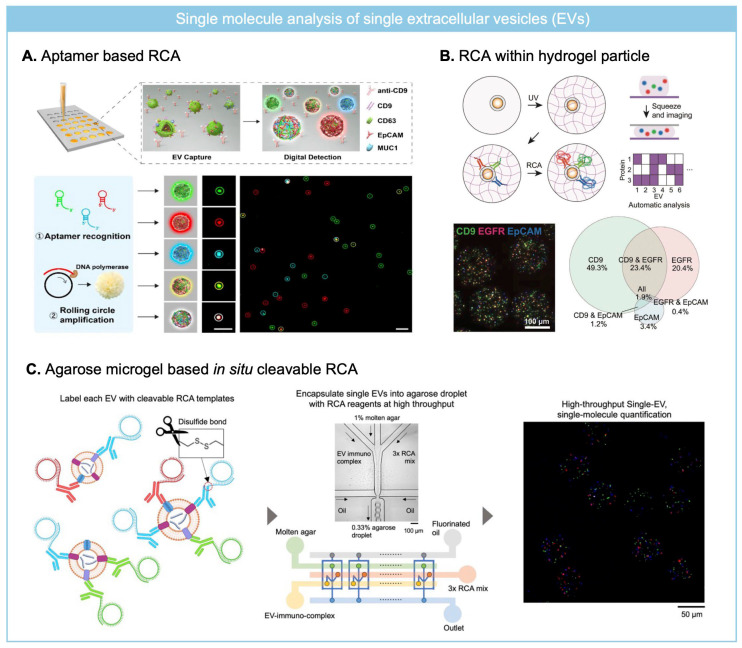
RCA for molecular cargo profiling of single EVs at the single-molecule level. (**A**) Aptamer-based RCA for analyzing multiple surface protein of individual EVs captured on a glass substrate. Reprinted/adapted with permission from Ref. [72]. Copyright 2020, John and Wiley Sons. (**B**) MeHA hydrogel particles were used for capturing single EVs, and multiplexed immuno-RCA was employed for multiplexed surface protein profiling. Reprinted/adapted with permission from Ref. [73]. Copyright 2025, John and Wiley Sons. (**C**) Agarose droplet based in situ cleavable RCA was employed to quantify multiple surface protein expression level at the single molecule level from single EVs. Reprinted/adapted with permission from Ref. [75]. Copyright 2025, American Chemical Society.

To amplify the signal from molecular cargo of single EVs, RCA-based assay has been widely adapted for single EV analysis where earlier approaches have focused on surface protein analysis. As an earlier approach, single EVs were captured on an antibody-coated glass substrate and three different surface proteins were labeled with aptamers including conjugation region for padlock probes (Figure 5A) [72]. And then, padlock probe is ligated followed by RCA reaction and RCA products are formulated over EVs.

Similarly, Roh et al. applied immune-RCA to analyze surface protein of single EV using a primer-labeled detection antibody. To overcome the spatial constraint of antibody access to captured EV on a substrate and facilitate sequential reaction on single EV required for signal amplification via RCA reaction, EV was trapped within hydrogel particles (Figure 5B) [73]. By capturing EVs within porous structure of hydrogel particles, repeated washing and reaction for RCA was enabled. For imaging RCA products over the EVs, hydrogel particles composed of methacrylated hyaluronic acid (MeHA) were squeezed to align RCA products labeled EV on a single focal plane. Four different surface markers were profiled on single EVs trapped within MeHA hydrogel particles, which revealed high heterogeneity depending on derived cell culture media. RCA-based single molecule analysis on single EV has relied on fluorescence imaging method, but single EV analysis requires high-throughput analysis to decrease the background noise signal so as to identify a rare population of EV from clinical specimens.

To overcome the low throughput of imaging, flow cytometry was employed to analyze RCA products formulated over EVs. As conventional flow cytometry can analyze particles larger than 200 nm, it has required aptamer triggered RCA reactions to form RCA products large enough to be analyzed by flow cytometry [74]. Surface protein of EVs is recognized by aptamer-guided RCA tailed-probes, and then RCA reaction is performed to form DNA aggregates over single EVs. With increasing RCA reaction time (up to 8 h), the size of RCA products over EV grew sufficiently large to be analyzed by flow cytometry. After the fluorescence labeling of RCA products over single EVs, it was detectable by both fluorescence imaging and flow cytometry. By targeting the dual surface protein (tetraspanin and tumor marker) of single EVs, it was able to distinguish EVs from different cell lines.

However, due to the large size of RCA products over single EVs, it has been difficult to quantitate surface protein at the single-molecule level. To overcome steric hindrance and crowding of RCA products over the EV, Park et al. developed an in situ cleavable RCA platform to resolve single molecule level surface protein on individual EVs (Figure 5C) [75]. The cleavage of RCA templates away from EV can overcome the steric hindrance of RCA products (>200 nm) over single EV (~100 nm) so that single molecule quantitation of single EVs can be achieved. EVs are labeled with primer-conjugated detection antibodies and unbound antibody conjugates are washed out using a size exclusion chromatography. Pre-circularized DNA is hybridized to DNA primer on EVs and then EV-RCA template complexes are encapsulated into agarose droplets with RCA mix including reducing agent under the digital regime. Disulfide bond included between DNA primer and antibody is cleaved once encapsulation into agarose droplet and RCA reaction is performed to amplify the signal from the surface protein of individual EVs. The amount of different fluorescent RCA products indicates different protein expression levels on single EVs, which are analyzable with flow cytometry as well as imaging. It was able to detect rare population of EVs with immune (PVR, PD-L1) or cancer-related markers (TYRP-1) from plasma samples of melanoma patients.

### 4.2. Internal Transcriptome

Furthermore, RCA can be used for amplifying the signal from internal miRNA within EVs; therefore, it has been employed for simultaneous profiling of surface protein and internal transcriptome [76]. Single EVs were captured by magnetic nanostirbar, enabling efficient sample preparation for RCA-based molecular cargo analysis. After EV fixation and cargo crosslinking on the nanostirbar, an aptamer for surface protein labeling and a DNA probe for miRNA targeting were applied to label the corresponding molecules for the subsequent RCA reaction. After the hybridization of circular DNA, RCA reaction, and fluorescence labeling of RCA products, EVs tethered on magnetic nanostirbar were loaded on glass for fluorescence imaging. They were able to count the ratio of EVs with miRNA for successful diagnosis of breast cancer patients at different stages.

## 5. Summary and Outlook

In this review, recent advances in applications of RCA-based single molecule assay have been overviewed including clinical specimens, single cell sub-cellular molecules, and single EV’s molecular cargo analysis (Table 1). Compared to representative ultra-sensitive detection platform, digital ELISA or digital PCR, RCA does not require compartmentalization, as it can localize the amplified signal via elongated single stranded DNA. Therefore, it is more beneficial for high-throughput analysis as it does not require loading into compartments under Poisson distribution (λ < 0.1) avoiding >1 bead per single compartment. Notably, it has been popularly employed for single cell sub-cellular molecules or single EV analysis to understand the inherent heterogeneity of biological systems by unveiling hierarchical structures in a multiplexed manner.

Although RCA-based single molecule counting assay has been widely adapted for various biological applications, there is still room for improving the sensitivity by decreasing the background noise signal or increasing the multiplex capability. DNA primer labeled antibody may induce higher amounts of non-specific binding compared to bare detection antibodies due to their electrical charge, which is a huge hurdle to decreasing background signal, thereby decreasing LOD. On the other hand, as it has shown in previous works, analyzing a larger sampling number can decrease the background signal represented by Poisson noise when non-specific binding is minimized. For substrate-based methods, large imaging area would be beneficial, and for bead-based approaches, analyzing a large number of beads is advantageous to achieving better performance of the assay. For increasing the multiplex capability, designing the sequence of circle DNA so as controlling the multiple fluorescence detection probes binding ratio to a single RCA product can overcome the spectral overlap of fluorescent dye [52,77]. Moreover, by applying artificial intelligence (AI)-driven signal analysis, the fluorescence ratio of RCA products can be determined with higher precision, thereby markedly enhancing both multiplexing capability and detection sensitivity [78]. In addition, AI-driven strategies for analyzing the hierarchical distribution of molecules within single cells or EVs in a high-throughput manner could further improve the overall accuracy of the assay [79].

There also remains limitations of RCA in terms of specificity and cost-effectiveness. For protein detection via immune-RCA, specificity is largely determined by the affinity of the antibody, and for nucleic acid detection via RCA, the specificity of probe recognition depends on complimentary sequence hybridization [80]. Although padlock probe-based RCA has been validated for single-nucleotide variation detection by positioning the variable nucleotide at the ligation site [81,82], challenges such as secondary structure formation and ligation fidelity must be addressed to achieve higher specificity. Furthermore, the high cost and thermal instability of phi29 polymerase remain critical barriers to clinical translation and commercialization. As a point of reference, nanozyme-based signal amplification strategies have shown promise in advancing the commercialization of ultrasensitive biosensing owing to their low cost and robustness [83,84].

With an enhancement in sensitivity, specificity, multiplex capability, and robustness, it is expected that RCA-based single molecule counting assay would be a universal tool for ultra-sensitive detection of biomolecules from various biological sources enabling diseases studying, diagnosis, or prognosis monitoring. Furthermore, it can be a high-precision point-of-care diagnostic platform by translating RCA-based single molecule counting assay with user-friendly lab-on-a-chip system [85].

## Figures and Tables

**Table 1:** Summary of RCA-based single molecule counting assay for various applications.

Applications	Analysis Target	Target Molecules	Multiplex Capability	Substrate	Ref.
**Clinical specimen**	Protein	N protein of SARS-CoV-2	1	Antibody coated glass slide	[37]
	Protein	Brachyury (T-box transcription factor)	1	Antibody coated microbeads	[41]
	Protein	Cytokines (IFN-γ, IL-1β, IL-5, IL-6, IL-10, IL-12p70, IL-18, VEGF)	8	Antibody coated multi-fluorescent microbeads	[40]
	Nucleic acid	Virus (Ebola, Zika, Dengue) RNA	3	In solution (without substrate)	[43]
**Single cells**	Nucleic acid	mRNA (Tk1, MYC, STK15, ER, HER2, PR, Ki67, BCL2, VEGF)	9	Coverslip	[52]
	Protein + Nucleic acid	Protein (CD10, CD19, CD45), mRNA (e1a2, e13a2, e14a2)	6	Coverslip	[53]
	Nucleic acid	mRNA splicing variant (CD45RA, CD45RB, CD45RO)	3	Gelatin coated cover glass	[54]
	Nucleic acid	mRNA (ACTB, MYC)	1	Thiolated carboxymethyl HA microgel	[61]
	Nucleic acid	SNV of mtDNA	2	Collagen coated glass slide	[65]
	Nucleic acid	Large area deletion region of mtDNA	2	Agarose microgel	[56]
	Protein	Secreted antibody (IgG, IgM, IgA)	3	Coverslip	[66]
**Single EVs**	Protein	CD63, EpCAM, MUC1	3	Coverslip	[72]
	Protein	CD9, EGFR, EpCAM, MUC1	4	MeHA hydrogel microparticles	[73]
	Protein	CD63, PTK7	2	In solution (without substrate)	[74]
	Protein	Tetraspanin (CD9, CD63, CD81), PVR, PD-L1, TYRP-1	3	Agarose microgel	[75]
	Protein + Nucleic acid	CD63, miR-122	2	Antibody coated magnetic nanostirbar	[76]

## Data Availability

Data sharing is not applicable.

## Abbreviations

The following abbreviations are used in this manuscript:

RCA	Rolling circle amplification
FACS	Fluorescence activated cell sorting
HA	Hyaluronic acid
LAMP	Loop-mediated isothermal amplification
mtDNA	Mitochondrial DNA
NASBA	Nucleic acid sequence-based amplification
PCR	Polymerase chain reaction
RPA	Recombinase polymerase amplification
SNV	Single nucleotide variation
EV	Extracellular vesicle
AI	Artificial intelligence
APTES	(3-Aminopropyl)triethoxysilane
N	Nucleocapsid
CITE-seq	Cellular indexing of transcriptomes and epitopes by sequencing
Ig	Immunoglobulin
